# Exploring Suicide Pacts in the UAE: A Retrospective Case Series and Contextual Review

**DOI:** 10.7759/cureus.97481

**Published:** 2025-11-22

**Authors:** Rawdha H AlShamsi, Enas A Hanafi, Hamdah T Kalantar

**Affiliations:** 1 Forensic Medicine, Dubai Police - General Department of Forensic Sciences and Criminology, Dubai, ARE; 2 Medicine and Surgery, Mohammed Bin Rashid University of Medicine and Health Sciences (MBRU), Dubai, ARE

**Keywords:** caregiving burden, chronic illness, internet-facilitated suicide, mass suicides, mental health intervention, psychological autopsy, social isolation, suicide pacts, suicide prevention

## Abstract

Mass suicide, defined as two or more individuals ending their lives together either simultaneously or in sequence, represents an uncommon but important public health concern. Although global suicide rates remain high, collective suicides appear to be increasing and often arise from a combination of pressures such as financial hardship, psychiatric illness, and social dependency. This study aimed to explore this phenomenon by conducting a retrospective review of six cases recorded by the Forensic Medicine Department of Dubai Police between 2009 and 2013. Each case was analyzed through available forensic and circumstantial evidence, with inclusion limited to instances where clear mutual intent was demonstrated. The findings indicated that all six events involved elderly married couples, with methods including firearm use, carbon monoxide poisoning, and hanging. Suicide notes were present in most cases, while psychiatric history was infrequently documented. The results emphasize the vulnerability of older adults with physical or emotional dependency and highlight the need for targeted interventions in such populations.

This study aimed to describe the demographic, forensic, and psychosocial characteristics of suicide pact cases in Dubai and to contextualize them within existing literature. A retrospective case series review was conducted on six suicide pact cases (12 individuals) recorded by the Forensic Medicine Department of Dubai Police, UAE, between 2009 and 2013. Cases were included based on clear evidence of mutual consent, such as suicide notes or corroborating police and witness narratives. All cases involved elderly married couples (mean age 78 years). Methods included firearms (three cases), carbon monoxide poisoning, hanging, and drowning. Suicide notes were present in five of six cases, while psychiatric history was documented in only one individual. The findings highlight the vulnerability of older adults with physical or emotional dependency in this region. The reliance on external examinations and the lack of psychological autopsies are important limitations. This case series provides valuable regional data on a rare phenomenon and underscores the need for targeted psychosocial support for elderly couples and enhanced forensic investigation practices.

## Introduction

The term “suicide pact” entered the medical literature in 1961, when Cohen described it as “the decision of two or more individuals to end their lives together at the same location” [1]. Rosen, in 1981, outlined its distinctive features and emphasized recurrent patterns observed in documented cases [2]. Subsequent epidemiological work, including that of Brown and Barraclough in England and Wales, examined its prevalence and sought to clarify underlying causes [3]. This study aimed to describe the demographic, forensic, and psychosocial characteristics of suicide pact cases in Dubai and to contextualize them within existing literature. Suicide pacts are usually strongly motivated, frequently involving individuals who face unstable relationships or social isolation. They may take several forms, including family or collective suicides and dyadic suicides.

The latter remain rare, accounting for fewer than 1 % of all suicides and lack a universally accepted definition [4]. Schwartz et al. defined dyadic suicide as an agreement between two people to die together, whether simultaneously or sequentially, with some cases overlapping with homicide-suicide scenarios [5]. Brown, King and Barraclough proposed a classification of dyadic deaths according to the nature of the relationship and the motivational drive [6]. Planning is often concealed, with participants entering into agreements that are either simultaneous or sequential [7]. Major risk factors include advanced age, chronic illness, psychiatric disorders and previous suicide attempts [8]. Older married couples are particularly represented, though adolescent couples without children have also been reported [9]. Common psychological themes include depression, hopelessness, dependency and financial distress [2,8,10]. Suicide notes and the absence of external violence at autopsy help distinguish pacts from homicide-suicide events [1-3].

The influence of the internet on suicidal behavior has received growing attention. Studies in the early 2000s documented cases where strangers met online and arranged collective suicides [11,12]. This medium has broadened the scope of pact formation, facilitating agreements that extend beyond familial or intimate bonds.

Epidemiological analyses suggest that suicide pact victims are generally older than those who die by individual suicide, with couples forming the most common subgroup [3,13]. The male-to-female ratio in suicide pacts has been estimated at between 1:1 and 3:1, contrasting with the higher male predominance in solitary suicides [3]. Cross-national studies further indicate variation in older adult suicide rates, being higher in Western Europe and the United States than in countries such as Japan and India [14,15]. Motivating factors across settings include health deterioration, psychiatric illness, socioeconomic strain and relational conflict [8,13,16].

## Materials and methods

Study design

A retrospective case series was performed, reviewing all suicide pact cases documented by the Forensic Medicine Department of Dubai Police, United Arab Emirates, between January 1, 2009 and December 31, 2013.

Study population and sample size

The study included six suicide pact events (twelve individuals in total). Inclusion criteria required clear evidence of mutual consent, such as notes, testimonies, or corroborating circumstances. For this study, a suicide pact was defined as an agreement between two individuals to die together, established through clear evidence of mutual consent. This was determined by the presence of a joint suicide note, independent witness testimonies (e.g., from family members), or police investigation narratives that conclusively ruled out coercion or homicide. The distinction from homicide-suicide was based on the absence of defensive wounds, the method used, and the corroborating circumstantial evidence of mutual planning. Homicide-suicide cases were excluded.

Study measures

Data were extracted from case records, including scene inspections, autopsy reports (when performed), suicide notes, and witness testimonies. Variables documented were demographics, method of suicide, location, presence of psychiatric history, and contextual details. Data on nationality and socioeconomic status were not consistently available in the records and were therefore not analyzed.

Ethics statement

All data were anonymized. Ethical approval was obtained from the relevant institutional authority overseeing forensic research. Ethical approval for this retrospective study was granted by the Dubai Police General Headquarters Ethics Committee with a waiver for individual consent as the research involved anonymized archival data.

Statistical analysis

Descriptive analysis was conducted to summarize demographic and clinical characteristics. Frequencies, proportions, and mean values were reported. Comparative descriptive data were also compiled against published international studies (see Table 1).

**Table 1:** Comparative Characteristics of Suicide Pacts: UAE, Western Europe, and Japan

Characteristic	UAE (Current Study, n=6)	Western Europe (Brown et al. [3], Hunt et al. [10])	Japan (Ozawa-de Silva [15], Lee et al. [17])
Average age (years)	78 (range 71–85)	~50	~45
Relationship type	Married couples only	Predominantly couples, occasional friends	Couples and internet-based stranger groups
Gender ratio (Male:Female)	1:1	3:1	~1:1
Common methods	Firearms (67%), CO, drowning, hanging	Hanging, poisoning, overdose	Charcoal burning, hanging, overdoses
Presence of suicide notes	83% (5/6 cases)	60–70%	~50%
Psychiatric history	Rare (1/12 individuals, depression)	Common (up to 30–40%)	Rare
Internet involvement	Minimal	Moderate	High
Prevalence among all suicides	<1%	<1%	1–2%
Setting	Primarily private homes	Variable (homes, outdoors)	Homes, public places (incl. internet-arranged)

## Results

All six cases involved elderly married couples, with a mean age of 78 years (range: 71-85 years). Suicide notes were present in five of the six cases. Firearms accounted for most homicide-suicide events, while double suicides were carried out by carbon monoxide poisoning, submersion in a vehicle, and hanging. Five of the six incidents occurred in the couples’ own homes. A documented psychiatric history was noted in only one case, in which the husband had depression. In one event, the male partner survived for several days after the attempt, ultimately dying of starvation after refusing food. Another case highlighted loss of autonomy, with a disabled wife urging her husband to participate. In one instance, the husband alerted authorities before dying. Comprehensive toxicological screening was not performed in all cases; in the carbon monoxide poisoning case (Case 3), carboxyhemoglobin (COHb) levels were confirmed to be greater than 84% in both individuals, and the male partner also had a blood ethanol level of 0.24 g/L. For the other cases, including poisonings, specific toxicology results were not available. Detailed case summaries are provided in Table 2. These findings suggest that suicide pacts among elderly couples arise in the context of complex medical, social, and psychological burdens, often without a prior psychiatric diagnosis.

**Table 2:** Demographic and Details of Six Suicide Pact Cases in the UAE

Case	Age of Man	Age of Woman	Method of Suicide	Location	Autopsy	Psychiatric History	Details
1	81	85	Gunshot wound (firearm)	Near home	No autopsy	None	Man survived initially, later died from starvation after refusing food. Suicide pact confirmed by written will.
2	78	79	Vehicle submerged (drowning)	Car in water	External examination	None	Husband took wife for a drive, later found submerged. A message led authorities to the scene.
3	78	73	Carbon monoxide (vehicle)	Garage	No autopsy	None	Suicide note found with instructions about wills and organ donation refusal. High carboxyhemoglobin levels in both victims.
4	72	71	Hanging (rope)	Garden hut	No autopsy	None	Both found hanging with feet touching the ground. Suicide note at the scene.
5	81	84	Gunshot wounds (firearm)	Bedroom	No autopsy	Depression (husband)	Woman had physical impairment, begged husband to end their lives. Suicide note at the scene.
6	78	80	Gunshot wounds (firearm)	House patio	No autopsy	None	Man called the police to confess, then shot his wife and himself. Suicide note found.

Table 1 provides a descriptive comparison based on the current UAE case series and selected international literature. The small sample size of the UAE series precludes statistical generalization, and the comparisons are intended for contextual purposes only. Compared with Western Europe and Japan, suicide pacts in the UAE involved markedly older participants, exclusively elderly married couples, and relied predominantly on firearms. Documented psychiatric histories were rare, in contrast to the higher prevalence reported in Western cohorts. Furthermore, internet facilitation, which is prominent in Japan, was not observed in the UAE series. 

## Discussion

This review identified six suicide pacts, involving 12 deaths. A pact can only be confirmed when evidence of mutual consent is present, such as a written note or clear inquest findings. Double suicides by hanging or carbon monoxide poisoning were the most frequent methods, whereas events involving firearms presented greater difficulty in distinguishing pacts from homicide-suicide. Without a note or corroborating evidence, establishing shared intent remains problematic [17,18].

Firearms were used in several cases [1,5,12]. This may reflect accessibility within the regional context, consistent with earlier studies reporting similar patterns [1,5]. Risk factors identified in prior research, including advanced age, chronic illness and psychiatric history, were also apparent in this series [3,12]. All couples in our study were elderly, with a mean age of 78 years, a figure notably higher than the average age reported in earlier European and Japanese studies [1,5,9,18,19]. The predominance of older couples in the present series underlines the role of aging, dependency and shared loss of autonomy in pact formation. Loneliness and social isolation are also documented as contributory factors in elderly suicides [18,19].

Brown and Barraclough (1999) [9] noted the existence of suicide pacts among younger couples without children, but no such cases were encountered in our study. This distinction suggests cultural and demographic differences in the profile of pact suicides in the UAE, where marital status, long-term dependency and advanced age appear central. However, it is important to note that with only six cases, these patterns may reflect random clustering rather than a definitive national trend. These findings support the need for interventions specifically directed at older adults, particularly those with chronic illness or caregiving responsibilities.

In each case in this series, the verdict was a suicide pact. Investigations relied mainly on external examination, with autopsy seldom requested. The omission of full autopsies in the majority of cases is a significant limitation, as it reduces diagnostic certainty regarding the cause of death and the ability to fully exclude underlying natural disease or subtle signs of coercion. This practice is often influenced by local jurisdictional procedures and religious considerations, but it inherently limits the forensic validity of the findings. Psychiatric histories were rarely documented and psychological autopsies were not undertaken, often for financial or procedural reasons. Legal inquiries are primarily concerned with excluding homicide rather than exploring psychosocial determinants [9,11,13]. This limits understanding of underlying motives. In the UAE context, where suicide is highly stigmatized and influenced by religious prohibitions, engagement with mental health services is low, which likely contributes to the underdiagnosis of psychiatric conditions, as seen in our series.

The rarity of these events and the restricted geographical setting constitute clear limitations. Yet, the findings emphasize the importance of training medico-legal and mental health professionals to identify warning signs in at-risk couples, especially where dependency or institutionalization is present. Whether or not consent is demonstrable, careful assessment and early intervention are essential. The application of psychological autopsies, although rarely undertaken in countries such as France or Japan, could offer valuable insights into intent and relational dynamics. Broader use of such methods may substantially improve our knowledge of the psychosocial and cultural contexts that shape suicide pacts [17,20].

The demographic profile of suicidal deaths, including factors like age and gender, has been explored in other regions, such as in a study from North India by Nagar and Bastia (2022) [21], which provides a comparative perspective on hanging deaths. While their study focused on individual suicides, it underscores the importance of regional demographic and psychosocial data in understanding suicide patterns. Table 1 compares demographic patterns, methods, and psychosocial features across the UAE, Western Europe, and Japan.

The literature on suicide pacts highlights their heterogeneity across demographic, clinical, and sociocultural dimensions. Case reports from Japan and Croatia have highlighted rare but instructive double-suicide methods, including intravenous potassium administration following psychopharmaceutical intake [22] and other lethal poisonings [23]. A forensic review of six French cases demonstrated that suicide pacts often involve intimate partners, with firearms commonly used, suicide notes frequently present, and challenges in differentiating consensual acts from disguised homicide-suicides [24]. Haenel and Elsasser (2000) similarly described double suicides and homicide-suicides in Switzerland, noting that careful forensic investigation is essential to distinguish between consensual and non-consensual deaths [25]. More recent work indicates a diversification of pacts through the internet and social media, broadening victim profiles and necessitating multidisciplinary prevention strategies [26]. Large-scale registry data from the United States confirmed two major pact types, self-harm and assisted suicide, with differing demographics and methods [27]. A comparative NVDRS analysis further distinguished pacts from solitary suicides and homicide-suicides [28]. Similarly, a detailed report of a suicide pact by hanging emphasized the importance of documenting method and context to differentiate pacts from other fatal events [29].

Clinical surveys reinforce the psychiatric dimension of pacts. In England and Wales, nearly 30% of identified pact cases had recent mental health service contact [13]. Earlier epidemiological studies identified married older couples as the predominant group, typically relying on passive methods such as poisoning or exhaust inhalation, with half of the victims affected by mental disorders and a third by physical illness [9]. The advent of internet-mediated suicide pacts has introduced new risks, particularly among strangers forming alliances online [8]. Finally, population-based data from the UAE reveal that suicidal behavior among adolescents is prevalent, with anxiety, loneliness, and tobacco use significantly associated [30].

We recognize certain limitations in our study that may influence the interpretation of the results. This study is limited by its small sample size, restricted geographical scope, and reliance on retrospective forensic records. Lack of systematic psychiatric documentation and the absence of psychological autopsies restricted the ability to fully explore underlying motives. In addition, reliance on external examination rather than complete autopsy in most cases reduced clinical details. The primary limitation is the reliance on external examinations without full autopsies in five of the six cases, which fundamentally affects the diagnostic certainty of the cause of death and the ability to definitively exclude homicide or underlying natural disease. The absence of comprehensive toxicological data in most cases, including the poisoning and drowning cases, further weakens the forensic validity. The small number of cases precludes generalization and the observed demographic patterns may be due to chance. Furthermore, crucial sociocultural variables such as socioeconomic status, religious background, and nationality (UAE citizens vs. expatriates) were not available for analysis, limiting the interpretation of findings within the specific Gulf context.

## Conclusions

This case series and literature review highlight the complex interaction of psychological, social and circumstantial factors in mass suicides and suicide pacts. Analysis of six suicide pact cases revealed a predominant pattern among elderly married couples with physical or emotional dependency. Proactive mental health intervention is especially critical in older adults experiencing chronic illness, social isolation, or caregiving burden. The growing role of technology, particularly the Internet, has introduced new pathways for arranging suicide pacts. Future research should incorporate extended psychological autopsies to better understand motives and mental states, alongside community-based approaches and mental health education. Emerging tools in behavioral diagnostics may provide opportunities for earlier detection and prevention.

A notable proportion of cases occurred without documented psychiatric history, suggesting underdiagnosis or lack of engagement with healthcare. This underscores the importance of routine mental health screening in at-risk populations. Prevention requires a multidisciplinary approach that integrates medical, legal and technological strategies. Although rare, suicide pacts carry significant public health implications and addressing both technological and psychosocial drivers is essential for effective intervention. Future prevention efforts should focus on community-based mental health support for elderly couples and enhanced forensic practices, including psychological autopsies where feasible.
